# Intraoperative Cerebellar Ischemic Stroke During Scoliosis Correction Surgery in a Pediatric Patient: A Case Report

**DOI:** 10.7759/cureus.95082

**Published:** 2025-10-21

**Authors:** Juan J Coba-Lozano, Juan F Vargas-Moreno, Frank M Herrera-Méndez

**Affiliations:** 1 Research, Instituto Roosevelt, Bogotá, COL; 2 General Medicine, Instituto Roosevelt, Bogotá, COL; 3 Spine Surgery, Instituto Roosevelt, Bogotá, COL

**Keywords:** case report, cerebrovascular accident, complications, ischemic stroke, pediatrics, spinal fusion

## Abstract

We present the case of a 14-year-old female patient with cerebral palsy, spastic hemiparesis, and neuromuscular scoliosis. Her medical history includes scoliosis managed with a growing rod lengthening system in 2020 (at age 9), followed by a spinal lengthening procedure one year later. The patient underwent scoliosis correction surgery in 2025. During the procedure, she experienced a loss of somatosensory evoked potentials (SSEP) and motor evoked potentials (MEP). A subsequent postoperative brain magnetic resonance imaging (MRI) revealed a cerebellar ischemic stroke, contrasting with a normal preoperative brain MRI. Despite this, she showed no functional or neurological deterioration compared to her baseline status. This case is notable for the occurrence of the cerebrovascular accident (CVA) during surgery, which prompted investigation into potential causes, such as global hypoperfusion by monitoring intraoperative vital signs. It also highlights the importance of rigorous monitoring and multidisciplinary communication during scoliosis surgery and suggests the need for prospective studies to evaluate the incidence and risk factors for intraoperative stroke in pediatric patients.

## Introduction

Surgical correction of scoliosis can dramatically improve a patient's quality of life, the procedure carries significant risks that demand meticulous preoperative planning, intraoperative monitoring, and postoperative care; however, it also entails a series of risks that the surgeon and their multidisciplinary team must be aware of to minimize them [1]. Within this series of risks, one that is poorly documented in the scientific literature, but which can cause devastating and irreversible consequences for pediatric patients, is the cerebrovascular accident (CVA) [2].

Previous case reports describing this type of event are scarce, and this complication is not frequently recorded in neuromuscular scoliosis surgeries in the pediatric population [3,4]. The reviewed literature reports a stroke incidence of 0.57% in this population, with a mortality rate of up to 7.6% [5,6]. These reports describe the presentation of stroke in the postoperative period [3,5,7]. However, no records were found regarding the occurrence of this event during the intraoperative period, as observed in the present case.

## Case presentation

We present the case of a 14-year-old female patient with a history of cerebral palsy, classified as level I in the Gross Motor Function Classification System (GMFCS), manifesting as right spastic hemiplegia and secondary neuromuscular scoliosis. The diagnosis of cerebral palsy was established at age 2, supported by brain magnetic resonance imaging (MRI), which revealed hyperintense lesions in the white matter, secondary to perinatal hypoxia. Despite these neurological findings, she demonstrated age-appropriate academic performance, without the need for neurodevelopmental therapy.

Her history included scoliosis management with a growing rod system, implanted in 2020 (at age 9), with a single subsequent lengthening performed a year later without complications. The patient, accompanied by her primary care provider, consulted the spinal surgery service in 2024 (at age 14) to resume the surgical plan for spinal lengthening.

**Figure 1:**
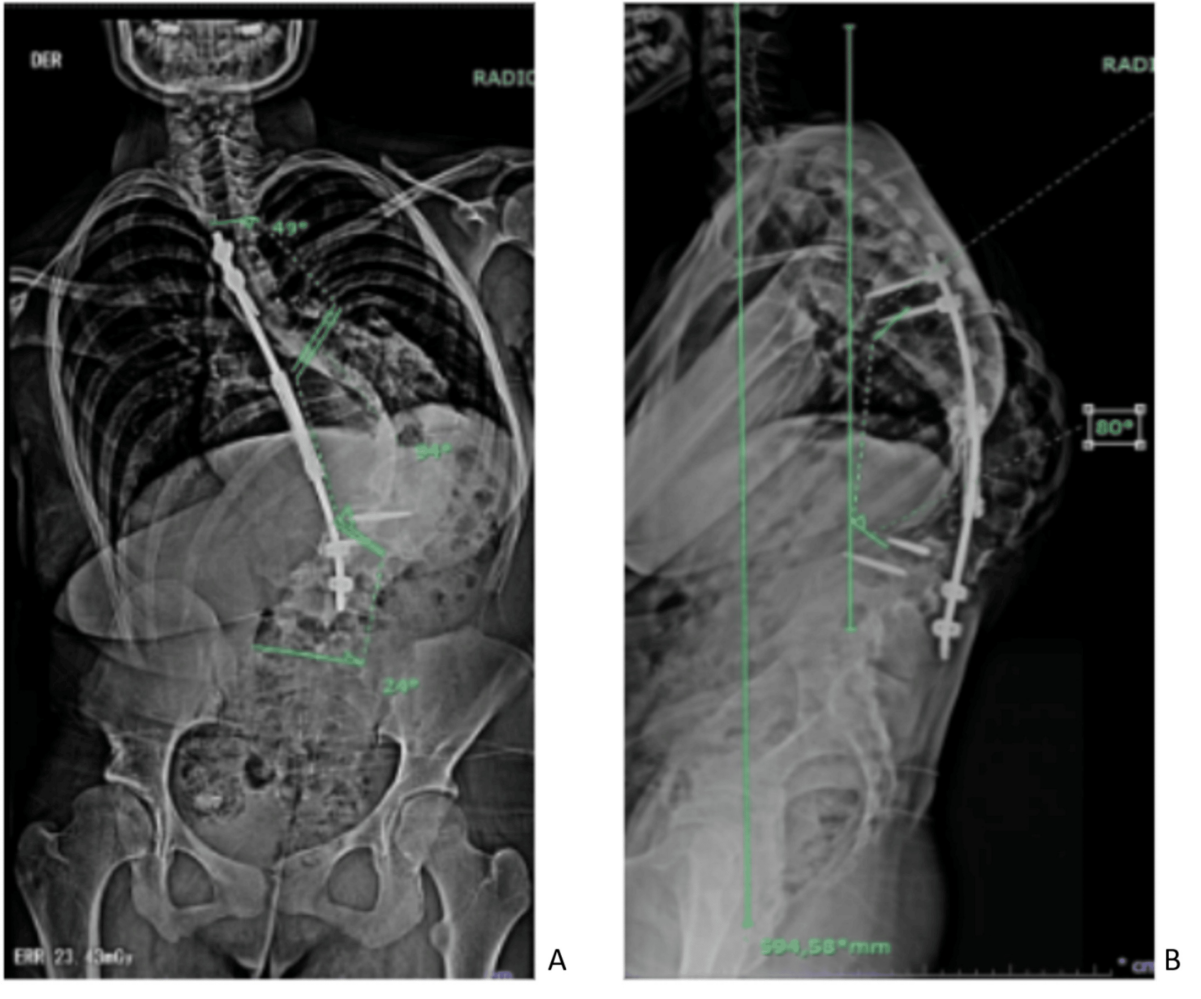
Preoperative anteroposterior (AP) and lateral panoramic spinal radiographs obtained 12 months before surgery. (A) Proximal thoracic curve measuring 49°, main thoracic curve 94°, and thoracolumbar curve 24°, demonstrating right coronal imbalance. (B) Mechanical failure of the lengthening system due to fracture of the distal transpedicular screws. Thoracic kyphosis from T5 to T12 measured 80°, with positive sagittal balance.

During the spinal surgery consultation, the patient reported occasional pain in the thoracolumbar and rib regions, with an intensity of 4/10 on the Numerical Rating Scale (NRS). Consequently, a panoramic spine X-ray was performed (Figure 1), which revealed a fracture of the lumbar transpedicular screws. She was determined not to be a candidate for further lengthening.

She had already reached menarche over two years earlier, so surgical treatment for scoliosis correction was planned in two stages under intraoperative neurophysiological monitoring: a first stage for removal of the lengthening material, and a second stage for placement of transpedicular rods and screws to achieve definitive correction. The patient and her family had no other significant medical, genetic, or psychosocial history.

**Figure 2:**
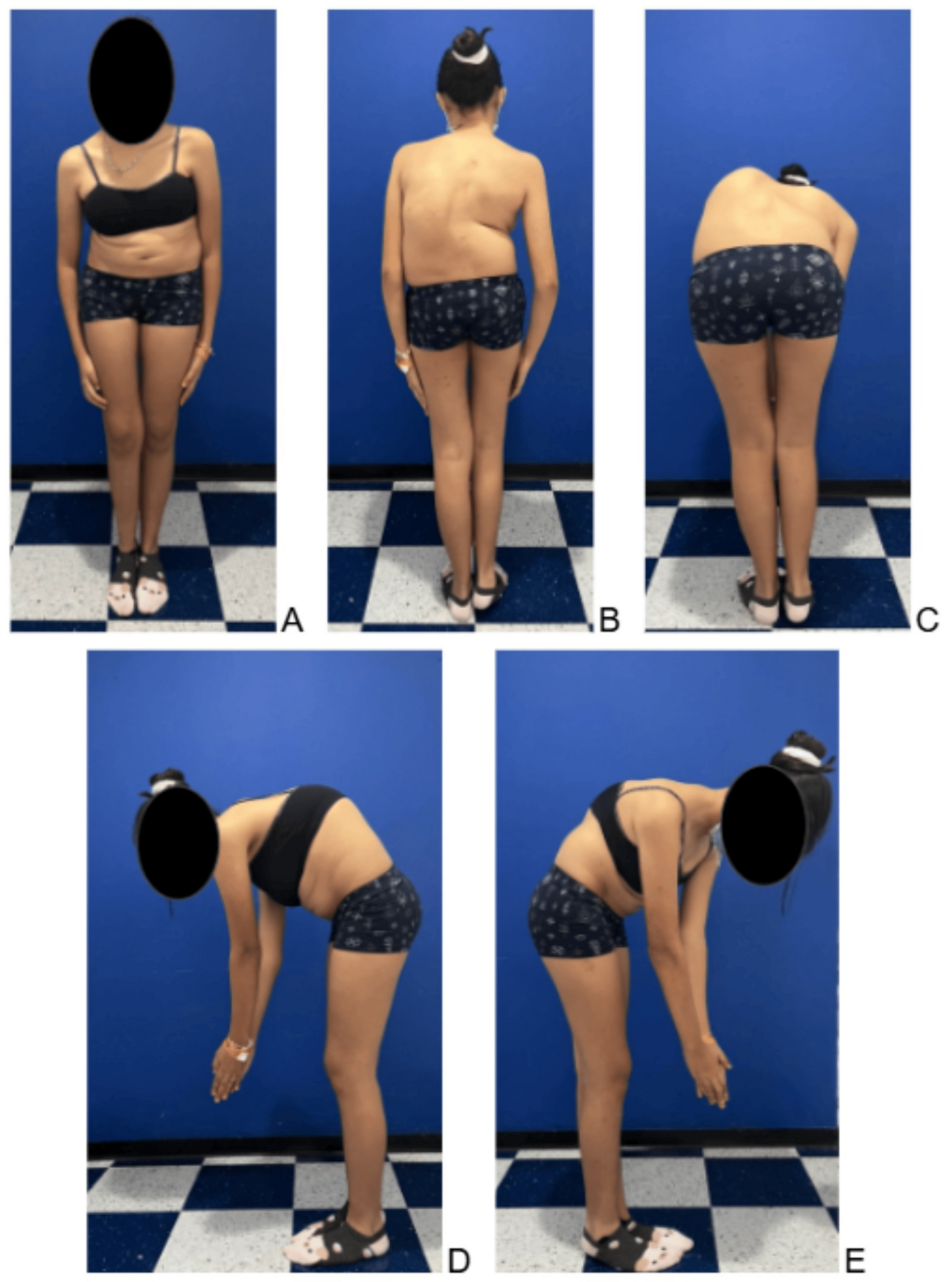
Preoperative clinical photographs taken eight months before surgery. (A) Anterior view in the standing position. (B) Posterior view in the standing position, showing right coronal imbalance and a right paravertebral sulcus. (C) Posterior view during forward flexion (Adams test). (D, E) Left and right lateral flexion views demonstrating marked thoracic hyperkyphosis.

The patient was admitted to the medical center in April 2025 (age 14) to carry out the established surgical plan. Initial physical examination revealed a hemiparetic gait with pronounced dysmetria of the right lower extremity, a rigid left thoracolumbar scoliotic curve, a positive Adams forward bend test with an ipsilateral 5-cm thoracolumbar rib hump, and left shoulder elevation. She had a rigid right pelvic obliquity, and the surgical wound from the prior spinal procedure was in good condition (Figure 2).

The neurological examination revealed bilateral patellar hyperreflexia with associated right ankle clonus, a positive Babinski sign on the right, and a negative Babinski sign on the left. Furthermore, biceps and triceps reflexes were bilaterally hyperreflexic, accompanied by a positive Hoffmann’s sign. The abdominal cutaneous reflex remained intact. Motor strength was assessed using the Medical Research Council (MRC) scale (also known as the Daniels scale), revealing right-sided hemiparesis graded 4/5, while the left side demonstrated full strength (5/5). Sensation was grossly intact throughout the body.

The patient underwent the first scoliosis correction surgical stage, during which the lumbar construct mechanical failure, seen in the X-ray, was confirmed, leading to the non-complicated removal of the broken instrumentation. The patient remained hospitalized between stages, following the medical center protocols and presented no complications. One week later, she was taken to the second surgical stage. During the preparation of the pedicle tract on the convex side of the curve, specifically during the insertion of the first transpedicular screws, there was a sudden loss of somatosensory evoked potentials (SSEP) and motor evoked potentials (MEP) bilaterally in the lower extremities.

The anesthesiology team performed the Stagnara wake-up test, which was negative, showing no voluntary movement of the lower limbs. Consequently, the recently placed instrumentation was removed, after which partial recovery of somatosensory potentials was observed. The wound was closed in layers, and the procedure was terminated.

**Figure 3:**
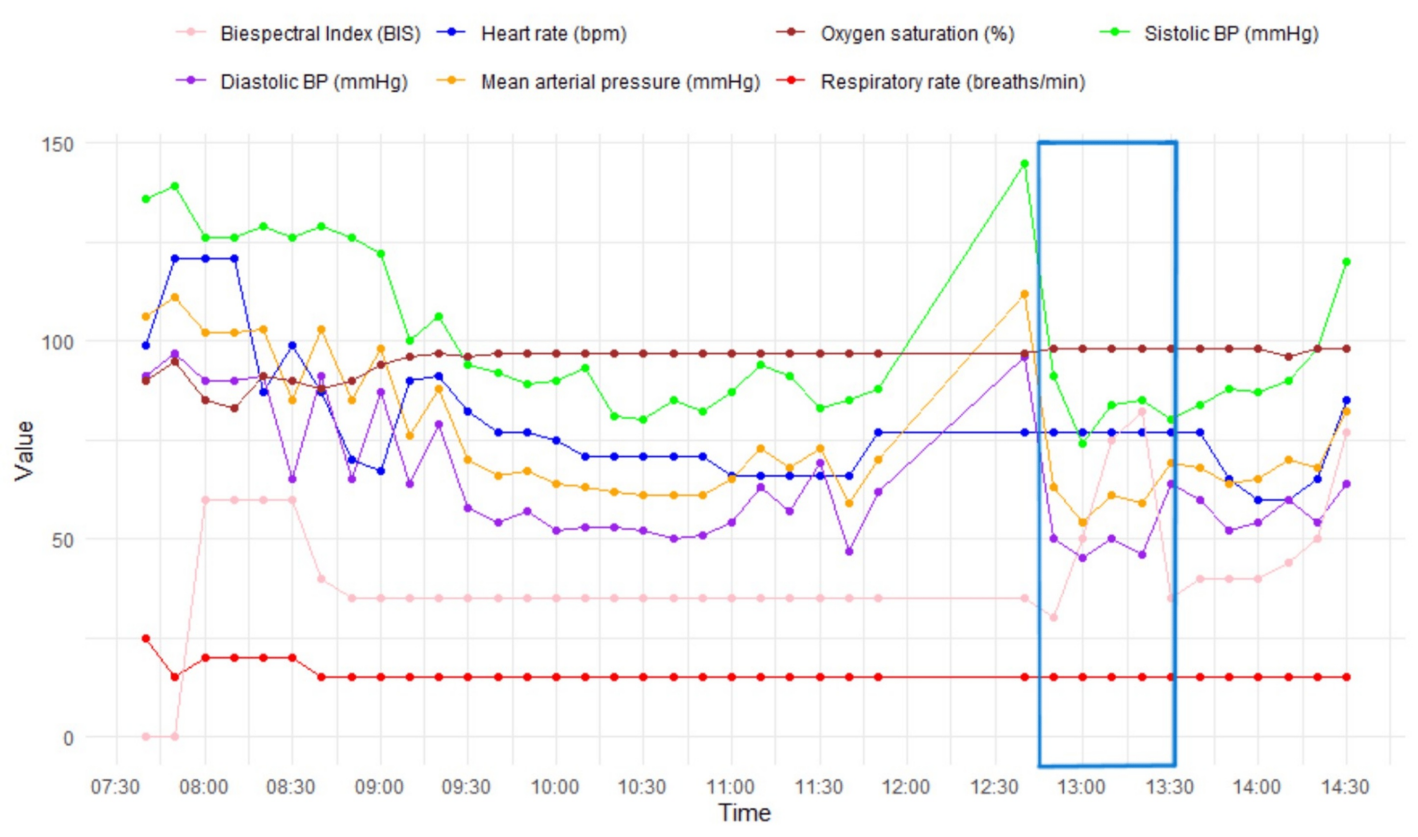
Intraoperative vital sign records. Periods of intraoperative hypotension was noted, with a transient hypertensive spike corresponding to the presumed event of neurological change. The Stagnara wake-up test (blue box) revealed increased posterior intraoperative brain activity. No vital signs were recorded between 12:00 and 12:30. BP, blood pressure; bpm, beats per minute; mmHg, millimeters of mercury; breaths/min, breaths per minute

According to the medical center's protocol, the patient was transferred to the intensive care unit (ICU) for close postoperative neurological monitoring. It should be noted that, in the intraoperative period, the patient presented moments of hypotension with mean arterial pressure (MAP) < 65 mmHg (Figure 3) that required the initiation of vasopressor support.

In the immediate postoperative period, the patient remained neurologically stable, showing no deterioration compared to her preoperative status and demonstrated voluntary movement in all four extremities. After two days in the ICU, she was transferred to the general ward, where new diagnostic imaging was requested -including a whole-spine computed tomography (CT) scan with 3D reconstruction and a whole-spine MRI - to assist in planning aimed at completing the second surgical stage for definitive scoliosis correction.

**Figure 4:**
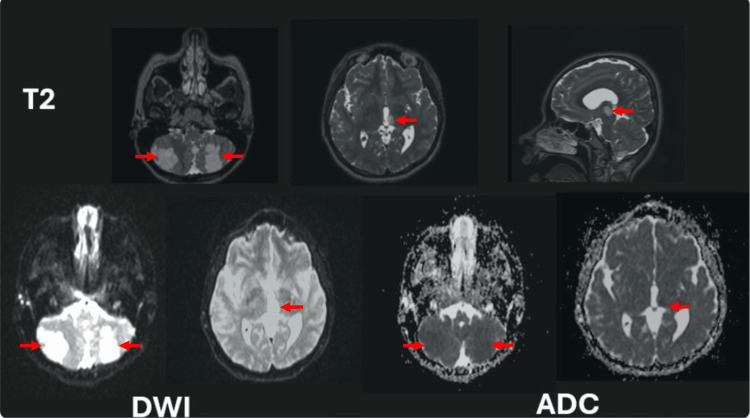
Brain magnetic resonance angiography (axial and sagittal T2, axial DWI, and ADC sequences). Multiple infarctions are observed in the left thalamus and both cerebellar hemispheres (red arrows), corresponding to the vascular territories of the left posterior cerebral artery (PCA) and the bilateral posterior inferior cerebellar arteries (PICA). DWI, diffusion-weighted imaging; ADC, apparent diffusion coefficient

The postoperative spinal MRI revealed areas of apparent ischemia in the cerebellum and thalamus, prompting consultations with pediatric neurology and neurosurgery. A brain magnetic resonance angiography was subsequently performed, demonstrating multiple acute infarcts involving the left thalamus and both cerebellar hemispheres, corresponding to the territories of the left posterior cerebral artery (PCA) and the bilateral posterior inferior cerebellar arteries (PICA) (Figure 4). The CT results showed no significant findings. To exclude an embolic etiology, the pediatric team ordered a Doppler ultrasound of the cervical vessels and a transthoracic echocardiogram, both of which were within normal limits.

Following multidisciplinary evaluation, the pediatric neurology service concluded that, based on current neuroimaging and an unchanged neurological examination relative to the preoperative status, a diagnosis of posterior reversible encephalopathy syndrome (PRES) could not be ruled out. They recommended continuation of rehabilitation and a repeat brain MRI in three to four weeks. The spinal surgery team determined that further corrective surgery was contraindicated due to the high risk of neurological deterioration.

A comprehensive rheumatologic workup, including homocysteine, rheumatoid factor, antiphospholipid profile, autoantibodies, antinuclear antibodies, and coagulation proteins C and S, was performed and found to be within normal limits. The rheumatology team found no evidence of autoinflammation or immunodeficiency disorders after reviewing the laboratory parameters. The patient was discharged 18 days postoperatively and referred for outpatient follow-up with pediatric hematology, neurology, physiatry, and spinal surgery.

At the time of preparing this case report, the patient remained clinically stable and did not have any new imaging control that could provide any additional information over the six-month follow-up period. There were no other unanticipated or undesired events associated with the procedure. No further interventions have been performed, and the patient is following the scheduled follow-up plan according to the medical center’s protocol.

## Discussion

This case describes a rare and serious complication - an intraoperative cerebellar ischemic stroke - occurring during neuromuscular scoliosis correction surgery in a pediatric patient. Complete preoperative records, anesthetic documentation, and diagnostic imaging enabled a thorough evaluation of possible contributing factors, and provided a comprehensive view of perioperative management. Although the surgery was performed in a national referral center with established scoliosis protocols, the absence of postoperative follow-up limits assessment of long-term neurological outcomes.

Arteriopathy accounts for up to 53% of pediatric strokes, yet the underlying mechanisms and risk factors remain incompletely understood [8]. Reported stroke incidence following neuromuscular scoliosis correction surgery is approximately 0.5%, with a mean patient age of 14 years (range: 2-37 years), and mortality reaching 7.6% in some series [5,6]. Most previously published cases occurred in the postoperative period and involved patients with neurological comorbidities such as neurofibromatosis type I or Chiari malformation type I [1,2,5].

This case exhibits several characteristics shared with other cases reported in the literature, including bilateral PICA involvement, a preexisting neurological deficit (spastic hemiparesis) [1,5], prone surgical position, and complete recovery with clinically insignificant sequelae [9].

A distinctive feature of this case is the timing of the onset of cerebellar ischemia. All stroke events have been described between one and 14 days postoperatively [5,10], being exceptional the presentation of the event in the intraoperative period. This allows an assessment of the possible causal factors of the ischemia and, in turn, the loss of SSEP and MEP.

Neuroimaging findings of bilateral cerebellar and thalamic infarctions, in the absence of cardiac or thromboembolic sources, suggest a multifactorial etiology. This is most consistent with selective hypoperfusion, possibly exacerbated by vertebral rotation maneuvers or embolic phenomena [11,12]. Although the hypothesis of global hypoperfusion was initially deemed unlikely due to hemodynamic stability and the absence of elevated lactate levels, a thorough analysis of intraoperative vital signs records revealed a decrease in cerebral perfusion pressure, leading to its re-evaluation and establishment as the primary causal hypothesis [13-15].

The pediatric neurology service also considered PRES as a differential diagnosis, characterized by cerebrovascular autoregulatory dysfunction associated with fluctuating blood pressure [9,12]. However, no prior reports have documented PRES following scoliosis correction surgery, making this diagnosis less likely.

## Conclusions

This clinical case emphasizes the importance of continuous intraoperative neurological monitoring, stringent hemodynamic control, and effective multidisciplinary communication during surgical correction of scoliosis. Although the patient experienced no new long-term neurological deficits, this event serves as a potent reminder for intraoperative cerebral ischemia, even in controlled surgical environments. Further prospective and multicenteric studies are essential to identify risk factors, refine preventive measures, and develop standardized protocols aimed at improving the safety and outcomes for pediatric patients undergoing scoliosis surgery.
